
JOURNAL INFORMATION
==============================
NLM Title Abbreviation: Sci China Life Sci
Journal ID: Sci China Life Sci
NLM Journal ID: 101529880

Science China. Life Sciences

ISSN: 1674-7305
EISSN: 1869-1889

ARTICLE INFORMATION
==============================
PMCID: PMC7089028
PMID: 25514849
DOI: 10.1007/s11427-014-4787-y
Article ID: 4787
Article version: 1
Subjects: News and Views

A new chapter for China’s public health security—aids offered to Africa to combat Ebola

Zhang BiKe
Gao George Fu gaofu@chinacdc.cn

grid.198530.6 0000000088032373 Chinese Center for Disease Control and Prevention, Beijing, 102206 China
Electronic publication date: 2014 Dec 16
Print publication date: 2015
Volume: 58
Issue: 1
First page: 114
Last page: 116
Received 2014 Nov 11; Accepted 2014 Nov 17
Copyright: © The Author(s) 2014
License: Open AccessThis article is distributed under the terms of the Creative Commons Attribution 4.0 International License (https://creativecommons.org/licenses/by/4.0), which permits use, duplication, adaptation, distribution, and reproduction in any medium or format, as long as you give appropriate credit to the original author(s) and the source, provide a link to the Creative Commons license, and indicate if changes were made.
License URL: https://creativecommons.org/licenses/by/4.0/

issue-copyright-statement: © Science in China Press and Springer-Verlag GmbH 2015

==============================

As one of the most deadly diseases, the outbreak of Ebola virus disease (EVD) is causing panic in West Africa and posing alarms globally. Since the re-emergence of EVD, China authority has been consistently providing support for Africa in its fight against Ebola. On September 16, 2014, a laboratory testing team and a medical team, consisting of 59 public health experts and medical staffers, were dispatched to assist local hospitals in West African countries to set up lab screening and clinical treatment of EVD (http://www.taiwan.cn/xwzx/Technology/201409/t20140917_7354545.htm). This action is not only a leap in concept, but also a step forward for China's public health security, exerting profound influence on social development and opening a new chapter for public health security in China [1,2].

## 1 The devastating Ebola epidemic

With rapid progress of globalization, infectious diseases in one region will eventually become a health crisis in other countries and regions [3]. The Ebola virus was first found in West Africa at 1970s. Since then, sporadic cases were reported with a small range of infected regions. Because of killing the host too quickly, Ebola was once considered as unsuccessful virus with limited capacity to spread by the international society [4]. In 2014, the re-emerging EVD has been spreading from rural areas to central cities in West African countries, with the trends to spread to more conjoint countries. As of October 29, 2014, there had been 13,567 reported Ebola cases with 4,941 reported deaths in eight affected countries, including Sierra Leone [5]. According to the World Health Organization (WHO), currently, epidemic statistics show that the situation in the three countries (Liberia, Sierra Leone, and Guinea) is not optimistic: the number of EVD patients has not declined till now. WHO predicted that the total number of EVD patients in Africa could rise to 20,000 by November 2014 [6]. According to the prediction by Centers for Disease Control and Prevention in the US, the number of EVD patients could reach 1,400,000 by January 2015 [7]. Early in August 2014, WHO announced this unusual West Africa EVD outbreak as "public health emergency of international concern" (http://news.xinhuanet.com/world/2014-08/08/c_1112001560.htm). The concern was then confirmed by the imported EVD cases in the US and Europe. This outbreak of EVD is "likely the greatest peacetime challenge" the United Nations has ever faced, said Dr. Margaret Chan, Director-General of WHO (http://news.xinhuanet.com/2014-08/02/c_126824461.htm).

## 2 China helps African countries to control Ebola

As of October 29, 2014, 523 medical staffers were infected by Ebola virus, resulting in 269 deaths, according to the WHO report on October 31 [6]. Due to the poor health facilities and weak medical health system in West African countries, it is difficult for them to deal with the Ebola epidemic and support from international community is desperately requested. Therefore, the Chinese government offered timely help and provided one million RMB worth of protective supplies to the three African countries in April. Till now, China has delivered four batches of supplies and provided funds with a total value of 750 million RMB (http://world.people.com.cn/n/2014/1031/c1002-25942380.html). More importantly, about 200 Chinese medical staffers have actively participated in the diagnosis and treatment of EVD under emergency conditions, when the local medical health system became half-paralyzed. This aid for African countries undoubtedly plays an important role in the containment of the Ebola outbreak. It also helps safeguard health of Chinese in African countries and lays foundation for future participation in international public health actions. Having an inside look at the Ebola epidemic, Chinese public health staff gained experience and learned Ebola prevention and control measures from international organizations. Thus, China has taken a step forward in preventing and controlling infectious diseases by offering help to African countries.

## 3 International communities speak highly of the Chinese medical teams in Sierra Leone

After arriving at Freetown, capital of Sierra Leone, on September 17, the Chinese laboratory testing team and medical team were put to the test in facing with the real threat of the Ebola virus and hand-on operations. The medical staffers were proud to witness several milestone events there: they reconstructed and upgraded the laboratory in Sierra Leone-China Friendship Hospital to a satisfactory testing laboratory within three days; using four testing reagents independently developed by China, they accomplished the testing of single blind samples from South Africa; the laboratory testing team analyzed 24 samples from Sierra Leone during the first testing day, which exceeded their target, 20 samples; the number of samples tested per day surpassed 100 for the first time on the day of October 31 (http://health.gov.sl/wp-content/uploads/2014/11/Ebola-Situation-Report_Vol-157.pdf); as of November 2, they analyzed 1205 samples in total, about 23.5% of samples in Sierra Leone. At the same time, they took advantage of Xiaotangshan Hospital's experience in dealing with SARS, and turned a general hospital into an infectious diseases hospital that is qualified to receive Ebola patients within a short period of time. Two hundred and fourteen observation cases were received and no medical staffers were infected till November 2. The performance of the Chinese laboratory testing team and medical team in Sierra Leone is very well received by international communities.

## 4 Active participation in global public health activities

Since the establishment of the nation, China has achieved remarkable success in control and prevention of infectious diseases and accumulated rich experience in coping with mass outbreak of epidemic. Over the years, China has been sending medical teams to Africa, but rarely participated in actions to cope with significant international public health emergencies, including epidemic. It lacks related concept, awareness, and experience, and a reliable platform for public health work outside China. By virtue of the framework of *International Health Regulations*, China should actively participate in international cooperation, shoulder the responsibilities and obligations, and strengthen mutual trust. Sharing rights, interests, and resources with the international community through communication of epidemiological information and technology, China will gain experience in coping with international public health events and in global control of related risk factors. In this way, cross-border spread of the diseases and risk factors can be prevented, reducing the risk for Chinese people.

## 5 Africa, one of the main public health battlefields for China in the future

The outbreak of EVD will, in a long period of time, affect health care in West African countries and other countries and regions in the world. In the action participated by various countries to help West African countries to combat Ebola, international cooperation in public health needs to be strengthened. Epidemic control is a global issue requiring strong professional backup, including experts specialized in related science and technology, especially medicine, and a public health system comprising effective disease diagnosis and treatment, disease surveillance, and public health intervention. Doctors have responsibilities to discover, report and treat infected patients; public health workers are to guide the government and people in control and prevention of various infectious diseases. An effective public health surveillance system needs to be established for timely collection, analysis and judgment of the situation. Epidemiological experts can carry out on-the-spot investigations, finding out reasons for the outbreak of the disease and proposing targeted intervening measures. Public education institutions have obligations to raise people's awareness towards infectious disease prevention. More public health professionals are needed to improve the implementation of strategies and measures for public health issues. Etiological experts can study the etiological characteristics of the disease and provide support for clinical diagnosis. All of these, only at the forefront of the epidemic, in every community and village, bear real significance. At present, African countries are fragile. They need more public health workers to go further to the frontline, get to know their demand and suffering, and guide them to prevent and combat the epidemic. At the meantime, public health workers themselves can gain more first-hand materials and experience in dealing with mass outbreak of such disease [2]. Thus, Africa is undoubtedly one of the main public health battlefields for China in the future.

**Open Access** This article is distributed under the terms of the Creative Commons Attribution License which permits any use, distribution, and reproduction in any medium, provided the original author(s) and source are credited.

This article is published with open access at link.springer.com


References

1 Gao GF, Feng Y. On the ground in Sierra Leone. Science, 31 October 2014, http://www.sciencemag.org/content/346/6209/666.full.pdf 10.1126/science.346.6209.666 25359978
2 Larson C. China ramps up efforts to combat Ebola. Science, 3 November 2014, http://news.sciencemag.org/asiapacific/2014/11/china-ramps-efforts-combat-ebola
3 Gong XQ Globalization of infectious diseases and global health care (in Chinese) Int Rev 2006 24 29
4 Li Y Ren X Liu D Cheng Y Gao GF Yu HJ Ebola virus disease: epidemiology, ecology, diagnosis, treatment, and control Sci Tech Rev 2014 32 15 24
5 World Health Organization. Ebola response roadmap update 31 October 2014. Geneva, Switzerland: World Health Organization, 2014
6 WHO Ebola Response Team. Ebola virus disease in West Africa—the first 9 months of the epidemic and forward projections N Engl J Med 2014 371 1481 1495 10.1056/NEJMoa1411100 25244186 PMC4235004
7 Meltzer MI Atkins CY Santibanez S Knust B Petersen BW Ervin ED Nichol ST Damon IK Washington ML Estimating the future number of cases in the Ebola epidemic—Liberia and Sierra Leone, 2014–2015 MMWR 2014 63 1 14 25254986
